# Epithelial‐mesenchymal transition softens head and neck cancer cells to facilitate migration in 3D environments

**DOI:** 10.1111/jcmm.13656

**Published:** 2018-05-04

**Authors:** Yin‐Quan Chen, Hsin‐Yi Lan, Yi‐Chang Wu, Wen‐Hao Yang, Arthur Chiou, Muh‐Hwa Yang

**Affiliations:** ^1^ Institute of Biophotonics National Yang‐Ming University Taipei Taiwan; ^2^ Biophotonics and Molecular Imaging Research Center National Yang‐Ming University Taipei Taiwan; ^3^ Institute of Clinical Medicine National Yang‐Ming University Taipei Taiwan; ^4^ Cancer Progression Center of Excellence National Yang‐Ming University Taipei Taiwan; ^5^ Division of Medical Oncology Department of Oncology Taipei Veterans General Hospital Taipei Taiwan

**Keywords:** cancer cells migration, epithelial‐mesenchymal transition, intracellular stiffness, Snail, three‐dimensional (3D) cell culture environments, Twist 1

## Abstract

The biological impact and signalling of epithelial‐mesenchymal transition (EMT) during cancer metastasis has been established. However, the changes in biophysical properties of cancer cells undergoing EMT remain elusive. Here, we measured, via video particle tracking microrheology, the intracellular stiffness of head and neck cancer cell lines with distinct EMT phenotypes. We also examined cells migration and invasiveness in different extracellular matrix architectures and EMT‐related signalling in these cell lines. Our results show that when cells were cultivated in three‐dimensional (3D) environments, the differences in cell morphology, migration speed, invasion capability and intracellular stiffness were more pronounced among different head and neck cancer cell lines with distinct EMT phenotypes than those cultivated in traditional plastic dishes and/or seated on top of a thick layer of collagen. An inverse correlation between intracellular stiffness and invasiveness in 3D culture was revealed. Knock‐down of the EMT regulator Twist1 or Snail or inhibition of Rac1 which is a downstream GTPase of Twist1 increased intracellular stiffness. These results indicate that the EMT regulators, Twist1 and Snail and the mediated signals play a critical role in reducing intracellular stiffness and enhancing cell migration in EMT to promote cancer cells invasion.

## 1 INTRODUCTION

Epithelial‐mesenchymal transition (EMT) is a process via which the epithelial cells lose their polarity and acquire the mesenchymal phenotype. EMT has been shown to be crucial in development, organ fibrosis and cancer metastasis.^1^, ^2^, ^3^ In cancer cells, the key features of EMT include a remodelling of cytoskeletons and a reduction in the intercellular adhesion between epithelial cells; concomitantly, the cellular morphology changes from cuboidal epithelial to elongated mesenchymal, which leads to enhanced migration & invasiveness and elevated resistance to apoptosis.^1^, ^2^ To date, most of the studies of EMT focus on its molecular and cellular biological aspects, including the signal transduction and the changes in biological behaviour. However, the corresponding changes in biophysical properties during EMT have attracted much less attention, and many important questions in the mechanobiology of EMT remain unanswered.

Cell mechanics plays an important role in determining the metastatic potential of cancer cells.^3^, ^4^, ^5^ Compared with the normal cells, the metastatic cancer cells are often softer, have less amount of cytoskeletons and are more invasive in trespassing through blood vessels.^6^ Recently, it has been reported that cell stiffness can serve as a biomarker of the metastatic potential of ovarian cancer cells.^7^ Most of the previous biomechanical studies focused mainly on cells cultured in non‐coated dishes, where cells are seated on a two‐dimensional (2D) glass substrate with stiffness on the order of 3GPa. The natural physiological environment of cells is, however, often three‐dimensional (3D), where cells are embedded in a complex dynamic environment of 3D extracellular matrixes (ECM)^8^ with stiffness in the range of tens of Pa to a few KPa, depending on the specific microenvironments.^9^

Extracellular matrix is an important mediator of cellular physiological and pathological functions.^10^, ^11^, ^12^ Depending on the specific physiological and pathological scenarios, cells sense different chemical and physical cues from the ECM (including its stiffness and architecture) via different signal transduction pathways to convert the physicochemical stimuli into biochemical signals^13^ and respond dynamically.^14^, ^15^, ^16^, ^17^, ^18^, ^19^, ^20^, ^21^ In general, the cellular responses may include changes in focal adhesion,^14^ cytoskeletal organization,^14^, ^16^ migration speed and trajectory,^19^ cellular differentiation,^17^, ^18^ proliferation^20^ and intracellular stiffness.^11^, ^22^ It has been well‐established that when cells are cultured in more rigid ECM, their focal adhesion, cytoskeletal organization and stiffness tend to increase.^11^, ^14^, ^16^, ^22^ In addition, the ECM microenvironment also regulates phenotypes of cancer cells. These studies imply that there is an intimate correlation between cell mechanics and cellular response to different ECM stiffness and architectures. However, the interplays among intracellular stiffness, geometry and stiffness of ECM and EMT progression have not yet been well characterized.

We previously demonstrated that the EMT regulator Twist1 induces mesenchymal migration of head and neck squamous cell carcinoma (HNSCC) cells through suppression of let‐7i to activate Rac1, and that the effect could only be observed in 3D cells culture.^23^ We therefore speculate that EMT‐induced cellular migration is associated with the changes in biophysical and biomechanical properties in 3D environment. In this study, we investigate the interplay between cell stiffness and EMT in HNSCC cells.

## 2 MATERIALS AND METHODS

### 2.1 Cell lines and shRNA experiments

The human head and neck squamous cell carcinoma (HNSCC) cell lines (FaDu, CAL‐27, SAS, OEC‐M1) were cultured in RPMI‐1064 supplemented with 10% FBS and 1% penicillin‐streptomycin solution at 37°C, 5% CO_2_. For shRNA experiments, we used two independent sequences to knock‐down Snail or Twist1 in OEC‐M1 or SAS cells. The first system for shRNA experiment was the pLKO.1 system. For gene silencing, pLKO.1‐shLuc, Snail shRNA (TRCN0000063819 and TRCN0000063821), and Twist1 shRNA (TRCN0000020540 and TRCN0000020542) were obtained from the National RNAi Core Facility of Taiwan. The second system for shRNA experiment was the pSUPER system. A hairpin sequence containing the shRNA against Snail or Twist1 was cloned into pSUPER.puro vector, and a scrambled sequence was used as a control. In OEC‐M1 cells, both pLKO.1 and pSUPER system were used. In SAS cells, we used pLKO.1 system with two independent sequences to knock‐down Snail or Twist1. The sequences of shRNA are given in Supplementary Table S1.

### 2.2 Culturing cells in different collagen matrix architectures

The 2D, 2.5D and 3D collagen type 1 matrices were prepared based on the method & procedure reported by Yang et al^23^ The concentration of collagen type 1 (ECM675, Millipore) is 1.71 mg/mL, and the corresponding matrix stiffness is ~259 Pa.

### 2.3 Video particle tracking microrheology

We applied video particle tracking microrheology (VPTM)^24^ to measure intracellular stiffness of HNSCC cell lines cultured in three different matrix architectures. Carboxylated polystyrene particles (Invitrogen, fluorescence excitation/admission peaks: 580/605 nm, diameter = 200 nm, concentration = 1.35 x 10^12^ particles/mL) were injected into the cells by a biolistic particle delivery system (PDS‐100, Bio‐Rad; pressure 450 psi). After particle injection, cells were washed with PBS twice and incubated for 4 hours. Then the particles‐bearing cells (approximately 10‐25 particles per cell) were cultured in different (2D, 2.5D and 3D) matrix architectures for 16 hours. Brownian motion of individual intracellular particles were recorded for 10 seconds via a fluorescence microscope (Nikon Eclipse Ti, Tokyo, Japan) equipped with a cell incubation chamber (TOKAI, Japan), an oil‐immersion objective lens (Nikon, 100x/N.A. = 1.45)*,* and a CMOS camera (Hamamatsu, Hamamatsu, Japan, OHCA‐Flash 4.0, 1024 × 1024 pixels), which enables us to record the images at a frame rate of 100 frames per second, and a spatial resolution of 0.13 *μ*m/pixel.

**Figure 1:**
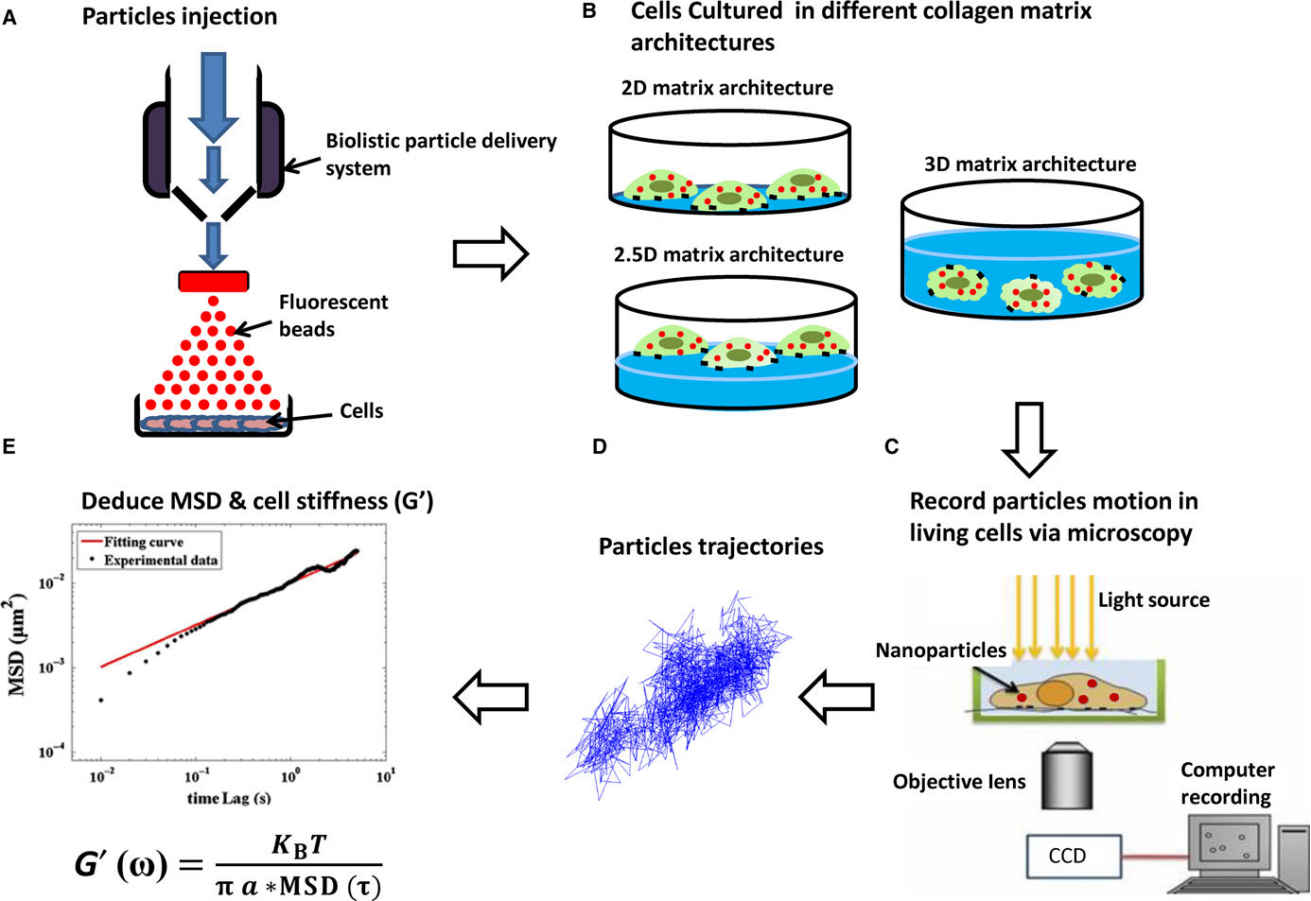
A schematic illustration of the measurement of intracellular stiffness in different extracellular matrix architectures based on video particle‐tracking microrheology. A, Carboxylated polystyrene particles (Invitrogen, fluorescence excitation/admission peaks: 580/605 nm, diameter = 200 nm, concentration = 1.35 x 10^12^ particles/mL) were injected into the cells by a biolistic particle delivery system (PDS‐100, Bio‐Rad; pressure 450 psi). B, After particle injection, cells were washed with PBS twice and incubated in different extracellular matrix architectures (2D, 2.5D, and 3D) for 16 h. C, Brownian motion of individual intracellular particles were recorded for 10 s via a fluorescence microscope equipped with a cell incubation chamber, an oil‐immersion objective lens (100x, NA = 1.45), and a CMOS camera, which enables us to record the images at a frame rate of 100 frames per second, and a spatial resolution of 0.13 μm/pixel. D, The two‐dimensional projection of the trajectories of the Brownian motion of each particle [*x*(*t*) and *y*(*t*), as a function of time (*t*)] were tracked and analysed. E, The ensemble‐averaged mean‐squared displacement (MSD), and the elastic modulus *G*′ (ω) were deduced from *x*(*t*) and *y*(*t*)

The two‐dimensional projection of the trajectories of the Brownian motion of each particle [*x*(*t*) and *y*(*t*), as a function of time (*t*)] were tracked and analysed via customized Matlab software (MathWorks, Natick, MA).^24^, ^25^ We calculated the ensemble‐averaged mean‐squared displacement (MSD), <Δ*r* ^2^(τ)> = <[*x*(*t *+ τ) − *x*(*t*)]^2^ + [*y*(*t *+ τ) − *y*(*t*)]^2^ >, the effective creep compliance *J*(τ) and the elastic modulus *G*′(ω) from the trajectory of each particle.^11^

(1) $$J\left(\tau \right)=\frac{\pi a}{K_{B}T}<\Delta r^{2}\left(\tau \right)>$$

(2) $$G^{\prime }\left(\omega \right)={\left[J(\tau )\right]}^{-1}$$

where “*a*” is the particle's radius, *K*_B_ the Boltzmann constant and *T* the absolute temperature. The intracellular stiffness (in Pascal, Pa) was measured and compared in terms of the value of the elastic modulus *G*′(ω) at frequency *f *= ω/2π = 10 Hz. Although our measurements allow us to determine *G*′(ω) in the frequency range of approximately 0.1‐100 Hz, the frequency of 10 Hz was chosen because at lower frequency, the experimental results may be affected by the noise due to any possible low‐frequency drift as well as the system 1/*f* noise, and the higher frequency is limited by the frame rate of the CMOS camera. Furthermore, 10 Hz is the typical frequency often used by many researchers in the cell mechanics community to compare the intracellular stiffness.^24^, ^25^, ^26^ A schematic illustration of our experimental procedure for the measurement of intracellular stiffness in different extracellular matrix architectures based on VPTM is given in Figure 1. Although VPTM provides not only the elastic modulus *G*′(ω), but also the viscous modulus *G*′(ω), we have deliberately excluded all the data related to the viscous modulus, because our results (not shown) indicate that, in the context of this paper, all the corresponding changes in *G*”(ω) are much less significant and also much less consistent.

### 2.4 Western blot analysis

For Western blots, cells were harvested and lysed in the protease inhibitor‐containing lysis buffer. The protein lysates were separated by SDS‐PAGE, transferred onto a PVDF membrane (Millipore Corp., Bedford, MA) and stained with proper antibodies. Signals were developed using an enhanced chemiluminescence kit (Millipore Corp., MA) and photographed with Fujifilm LAS‐4000. The antibodies used in this study include the antibodies against E‐cadherin (#4065, Cell Signaling Technology, Inc. Danvers, MA), vimentin (V6630, Sigma‐Aldrich Corp., St. Louis, MO), Snail (#17732, Abcam plc., Cambridge, UK) and Twist1 (#50581, Abcam plc., Cambridge, UK); and β‐actin (MAB1501, Chemicon International Inc., Billerica, MA) was used as a loading control. ImageJ software was used for densitometric measurements of the Western blots.

### 2.5 2D cell migration assay

A 8 μm Boyden chamber was used for migration assay. 10^5^ cells in medium containing 0.5% serum were seeded in the upper chamber, and 15% FBS was added to the medium in the lower chamber as the chemoattractant. After 16 hours, cells on the upper side of the filter were removed, and cells that remained adherent to the underside of the membrane were fixed in 4% formaldehyde and stained with Hoechst 33342 dye. The condition of 2.5D transwell invasion assay was the same as that of the 2D migration assay, except that the migration period was 24 hours and the inserts were coated with Matrigel.

### 2.6 3D invasion assay

Cells were plated for attachment, then the supernatant was removed and covered with collagen solution (1.7 mg/mL). 10% FBS‐containing medium was added as a chemo‐attractant, and the plates were incubated at 37°C, 5% CO_2_ for 24 hours. After incubation, cells were fixed with 4% formaldehyde and stained with Alexa‐488 coupled to phalloidin. A set of 2‐D image slices was collected in each well from the bottom of the well up to a height of 60 μm via a laser confocal microscope. The invasion index was calculated as the number of cells in 3D images reconstructed from 30 slices of 2‐D images from the depth of 31‐60 μm.

### 2.7 Cell mobility speed

Cells were allowed to adhere on 1.7 mg/mL collagen for 16 hours, then observed (and their images recorded every 10 minutes) for 24 hours in a humidified, CO_2_‐equilibrated chamber with a Leica DM IRBE microscope (Leica Microsystems Inc., IL). The trajectory of individual cells was tracked by MetaMorph^®^ software (Molecular Devices, Inc., CA).

### 2.8 Statistical analysis

Statistical significance of the experimental results was evaluated by Student's *t* test and indicated by * for *P* < 0.05 and ** for *P* < 0.01.

## 3 RESULTS

### 3.1 The epithelial‐type head and neck cancer cells exhibit larger increment in stiffness in 3D ECM architecture

**Figure 2:**
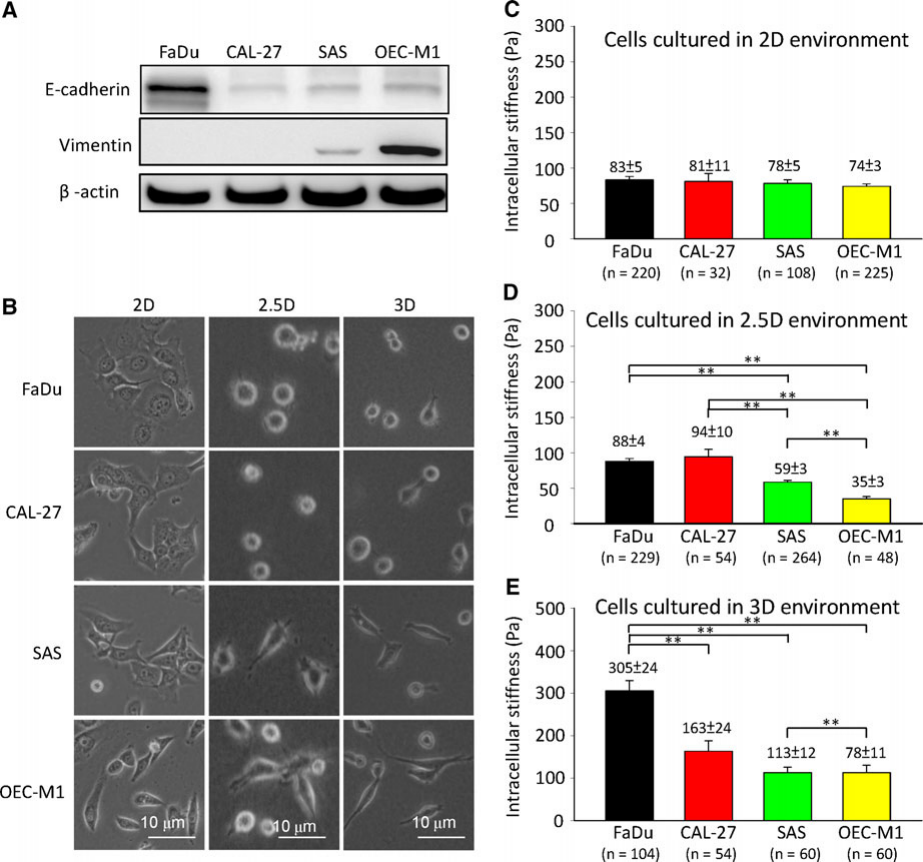
Extracellular matrix (ECM) architecture influences cell morphology and intracellular stiffness of HNSCC cell lines (FaDu, CAL‐27, SAS and OEC‐M1). A, Western blot of E‐cadherin and vimentin in four head and neck cancer cell lines FaDu, CAL‐27, SAS and OEC‐M1. β‐actin was used as a loading control. B, Phase contrast images of HNSCC cell lines cultured in 2D, 2.5D, and 3D environments. Scale bar = 10 μm. C‐E, The intracellular stiffness (at frequency *f* = 10 Hz) of HNSCC cell lines culture in 2D, 2.5D, and 3D environments. The numbers of cells are indicated in each panel. Data represent mean ± SEM **P < .01

To investigate the impact of EMT phenotypes and different ECM architectures on cellular stiffness in HNSCC cells, we measured the intracellular stiffness via video particle‐tracking microrheology (VPTM)^24^, ^25^, ^26^, ^27^, ^28^, ^29^ of HNSCC cells cultured in three different matrix architectures, including 2D (where cells were cultured on non‐coated glass dishes with a stiffness ~3 GPa), 2.5D (where cells were cultured on top of a thick layer ~190 μm of collagen type 1 with a stiffness ~259 Pa coated on glass dishes) and 3D (where cells were embedded in 3D collagen type 1 with a stiffness ~259 Pa)^23^ (Figure 1). VPTM enables us to measure the dynamic viscoelasticity, with sub‐cellular spatial resolution on the order of 1 μm, and with a frequency range ~0.1‐100 Hz, of living cells in different micro‐environments, including cells embedded in 3D ECM, which is rather challenging, if not impossible, via other techniques. Four HNSCC cell lines (FaDu, CAL‐27, SAS, and OEC‐M1) with well‐characterized EMT phenotypes were used in this study. In 2D culture, FaDu cells harbour the typical epithelial cells characteristics including a cobblestone‐like morphology and the expression of the epithelial marker E‐cadherin. In contrast, SAS and OEC‐M1 cells exhibit a mesenchymal phenotype including a fibroblastoid‐like morphology and the expression of the mesenchymal marker vimentin (Figure 2A,B). The morphology of cells cultured in 2.5D and 3D systems were distinct from the morphology in 2D: the epithelial‐type cancer cells showed a round morphology, whereas the mesenchymal‐type cells were elongated with protrusions; the differences were more pronounced in 3D environment (Figure 2B). However, the expression of the EMT markers (E‐cadherin, vimentin, Snail, and Twist1) in HNSCC cell lines cultured in 2.5D and 3D system were similar to those in 2D culture (Figure S1A). Besides, all four phenotypes of HNSCC cells cultured in 2D, 2.5D and 3D systems for 24 hours showed no significant differences in cell proliferation (Figure S1B).

Next, we investigated the intracellular stiffness of the four phenotypes of HNSCC cells cultivated in 3 different ECM architectures. Specifically, we applied VPTM to measure the intracellular stiffness (or elastic modulus), in the frequency range of approximately 0.1‐100 Hz, of HNSCC cell lines cultured in three different matrix architectures, including 2D, 2.5D and 3D. To facilitate the comparison, intracellular stiffness at the frequency of 10 Hz was used in this paper.

When cells were cultivated in 2D environment, the intracellular stiffness was in the range of 74‐83 Pa without significant differences among the 4 cell lines (Figure 2C). In 2.5D environment, the intracellular stiffness of mesenchymal‐type OEC‐M1 and SAS cells were significantly lower than that of the epithelial‐type cancer cells CAL‐27 and FaDu (Figure 2D). Interestingly, when cells were embedded in 3D architecture, the intracellular stiffness of all 4 types of cells (FaDu, CAL‐27, SAS, & OEC‐M1) increased by a factor of ~3.7, 2.0, 1.4, and 1.1, respectively, compared with the corresponding values in 2D ECM environment (Figure 2E). The increment in intracellular stiffness was significantly higher in epithelial‐type cells than in mesenchymal‐type cells (Figure 2C‐E) indicating that the intracellular stiffness of epithelial‐type cells was more sensitive to the different ECM architectures in comparison with the mesenchymal‐type.

### 3.2 An inverse correlation between cellular stiffness, EMT phenotype and migration capability in 3D environment

**Figure 3:**
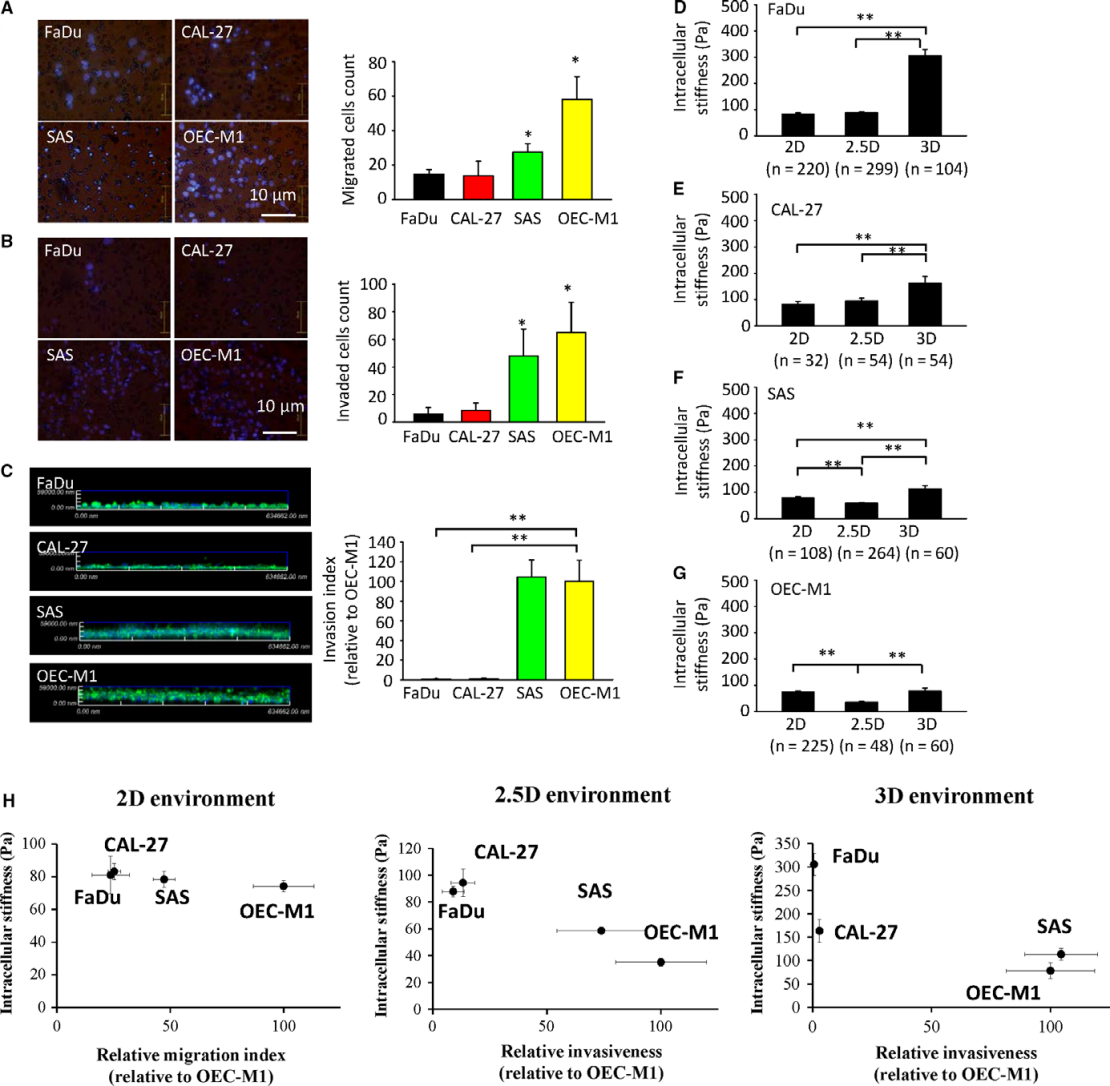
The migration capability and intracellular stiffness (at 10 Hz) of HNSCC cell lines in different extracellular matrix architectures. A, Left: Representative micrographs of the results of 2D transwell migration assays. Blue: nuclei staining. Right: quantification of the data. Data represent mean ± SEM n = 3. B, Left: Representative micrographs of the results of 2.5D transwell invasion assays (in which the insert was coated with Matrigel). Blue: nuclei staining. Right: quantification of the data. Data represent mean ± SEM n = 3. C, Left: Representative pictures of the results of 3D invasion assay. Data are presented as percentage of the invasion index of the control cells (OEC‐M1) indicated in each panel. Green: immunofluorescent staining of F‐actin of the cells. Right: quantification of the data. Data represent mean ± SEM n = 3. D‐G, The intracellular stiffness measured by video particle tracking microrheology (VPTM). H, The correlation between the relative migration capability (in the case of 2D), or relative invasiveness (in the cases of 2.5D and 3D), and intracellular stiffness of FaDu, CAL27, SAS, and OEC‐M1 cultured in 2D, 2.5D & 3D environments. *p < .05; **p < .01

Next, we investigated the effect of different ECM architectures on cellular migration and stiffness. For cell migration measurements, we used non‐coated transwells for 2D culture, Matrigel‐coated transwells for 2.5D system and collagen‐embedded wells for 3D. Our results show that the mesenchymal‐type cancer cells exhibited a higher migration capability than the epithelial‐type cells in all three systems. Notably, the differences were more pronounced in 3D system than in 2.5D and 2D. In 3D cells culture, the epithelial‐type cancer cells were relatively stiff and barely migrated; in contrast, the mesenchymal cells were relatively soft and highly migratory (Figure 3A‐C). Consistently, the increment in cell stiffness was also more pronounced in 3D compared with 2.5D and 2D, but only in epithelial‐type cells, and not in mesenchymal cells (Figure 3D‐G). A strong inverse correlation between EMT progression with invasiveness and intracellular stiffness of HNSCC cells was revealed in 2.5D & 3D environment (Figure 3H).

Taken together, these results reveal that the biomechanical, physical and chemical behaviours of cells cultured in 2D environment differ drastically from those in 3D; hence, cells culture in 3D could be an important first step towards a cell model system for better mimicking cellular behaviour in vivo.

### 3.3 Knock‐down of the EMT regulator Twist1 or Snail increases intracellular stiffness and reduces cell migration capability

**Figure 4:**
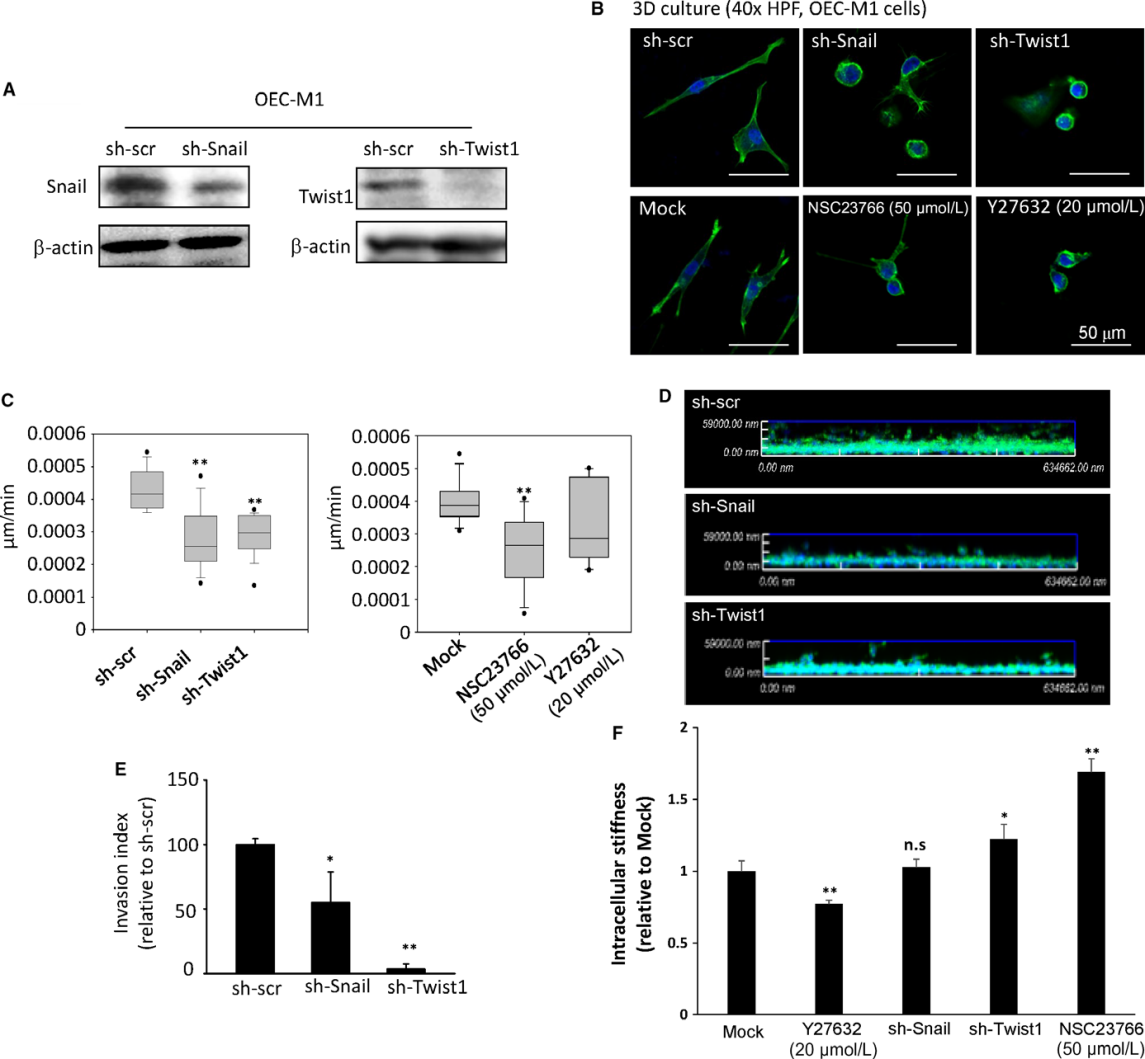
The effect of EMT signalling on cellular migration and intracellular stiffness. A, Western blot to show the expression of Snail and Twist1 in OEC‐M1 cells transfected with the pSUPER.puro vector containing a shRNA against Snail (sh‐Snail), Twist1 (sh‐Twist1), or a scrambled sequence (sh‐scr). β‐actin was a loading control. B, Immunofluorescence to show the morphology and actin organization of OEC‐M1 cells; upper panel: cells receiving sh‐Snail, sh‐Twist1, or sh‐scr; lower panel: cells treated with Rac1 inhibitor NSC23766 (50 μmol/L), ROCK inhibitor Y27632 (20 μmol/L), or mock. The cells were embedded in collagen (3D). Green, F‐actin; blue, nuclei. Scale bar = 50 μm. C, Quantification of motility speed of OEC‐M1 cells transfected with a shRNA against Snail, Twist1, or a scrambled sequence (left); treated with Rac1 inhibitor NSC23766 (50 μmol/L), ROCK inhibitor Y27632 (20 μmol/L), or mock (right)(n = 10 for each stable cell line). The cell motility speed was calculated and is presented as microns per minute. The box plots represent sample maximum (upper end of whisker), upper quartile (top of box), median (band in the box), lower quartile (bottom of box), and sample minimum (lower end of whisker). D‐E, 3D invasion assay. D, representative images of OEC‐M1 clones invaded into collagen after 24 h (n = 3). E, quantification of the invasion index relative to the control clones. Data represent mean ± SEM n = 3. F, A comparison of the relative intracellular stiffness (at 10 Hz) of OEC‐M1 cells cultured in 3D environment and transfected with a shRNA against Snail, Twist1, treated with Rac1 inhibitor NSC23766 (50 μmol/L), ROCK inhibitor Y‐27632 (20 μmol/L), or mock (normalized to that of the cells treated with Sh‐scr). Data represent mean ± SEM n = 3. *P < .05; **P < .01

Of the several possible molecular mechanisms in EMT progression, Twist1 has been identified as a master regulator of morphogenesis which plays an essential role in tumour metastasis.^30^, ^31^ We previously reported that Twist1 cooperates with BMI1 to suppress the expression of microRNA let‐7i, resulting in the activation of Rac1 and inducing mesenchymal movement in 3D environment.^31^ In this study, we observed that, in 3D culture environment, Rac1 activation of mesenchymal‐like HNSCC cells (SAS & OECM‐1) were significantly higher than that of epithelial‐type cells (FaDu & CAL27) (Figure S2). As the effect of the other major EMT inducer Snail on the migration capability of cells in 3D culture is unclear, and the impact of knock‐down of EMT inducers on intracellular stiffness is elusive, we further examined the effects of suppression of Twist1, Snail, Rac1 and Rho‐associated kinase (ROCK) activities on intracellular stiffness and migration of OEC‐M1 cultured in 3D environments. We used two independent shRNA systems (pSUPER, see Figure 4; and pLKO.1, see Figure S3) containing different sequences to knock‐down Snail or Twist1 in OEC‐M1 cells (Figures 4A and S3A,B). The wild‐type OEC‐M1 cells exhibited a mesenchymal morphology in 3D culture. Knock‐down of either Twist1 or Snail had a prominent effect in abrogating the elongated morphology, generated a round cell (Figures 4B and S3C) and reduced the activation of Rac1 (Figure S3D). Inhibition of Rac1 activity by NSC23766 or ROCK activity by Y27632 also partially attenuated the cellular protrusion and elongation (Figure 4B). In 2.5D system, knock‐down of either Snail or Twist1 or inhibition of Rac1 reduced the cell migration speed; the suppression of ROCK led to a similar, but smaller effect (Figures 4C and S3E). Likewise, in 3D environment, both Twist1 and Snail knockdown had a significant impact in reducing cell invasion (Figures 4D,E and S3F,G).

After confirming the impact of different EMT regulators and Rac1/ROCK inhibitors in HNSCC migration in 3D system, we investigated their impact on intracellular stiffness via the VPTM method. Our results showed a consistent increment of intracellular stiffness in OEC‐M1 cells receiving shRNA to knock‐down Twist1. Knockdown of Snail increased intracellular stiffness in one of the two clones (Figures 4F and S3H). Inhibition of Rac1 activity by Rac1 inhibitor NSC23766 also increased intracellular stiffness in a dose‐dependent manner (Figure S4A,B), but did not affect the phosphorylation of myosin light chain (Figure S4C), whereas the inhibition of ROCK activity induced a reduction in intracellular stiffness (Figure 4F). Furthermore, we investigated the role of myosin light chain (MLC) phosphorylation in cell invasion and intracellular stiffness of OEC‐M1 cultured in 3D environments. Our results showed that inhibition of myosin light chain phosphorylation by blebbistatin treatment resulted in decreased invasion capability (Figure S5A,B) and intracellular stiffness (Figure S5C). To further confirm the effect of EMT on cellular migration and intracellular stiffness in 3D environments, we used two independent shRNA sequences to suppress Twist1 or Snail in another mesenchymal‐type HNSCC cell line SAS. Consistently, our results showed that knock‐down of either Snail or Twist1 abrogated mesenchymal morphology, suppressed migration and invasion in 3D culture (Figure S6A‐E). Notably, suppression of either Twist1 or Snail caused an increment in intracellular stiffness of SAS cells; compared with Snail, Twist1 had a more prominent effect in SAS cells (Figure S6F). Conversely, we stably expressed Twist1 or Snail in epithelial‐type HNSCC cells (FaDu) cultured in 3D environments. Enforced either Twist1 or Snail expression in FaDu cells resulted in decreased intracellular stiffness (Figure S7).

## 4 DISCUSSION

The biomechanical and biophysical properties of cancer cells and the surrounding ECM modulate the metastatic capability of tumours. Most of the studies focus on the impact of ECM on cancer metastasis. Accumulated evidences support that tumour tissues are stiffer than healthy tissues, correlated with an increase in ECM stiffness through increasing collagen deposition and cross‐linking within tumour stroma.^32^, ^33^ In contrast, investigations of the intracellular stiffness of cancer cells during metastasis are relatively limited. A reduction in intracellular stiffness maybe helpful for cancer cells to deform and to penetrate through stiffer tumour stroma and endothelial cell‐cell junction in cancer metastasis.^5^ However, the change in intracellular stiffness during EMT is unclear. In this study, we analysed intracellular stiffness in four HNSCC cell lines with distinct EMT phenotypes and cultured in different extracellular matrix architectures to better understand the interplays among the cell mechanics, cellular external environment and EMT. In 3D ECM environments, the intracellular stiffness bears a strong inverse correlation with cell invasiveness and the expression of biomarkers of EMT. Among the four cell lines, the most mesenchymal phenotype, OEC‐M1, is the softest and the most invasive, whereas the most epithelial phenotype, FaDu, is the stiffest and the least invasive. Knock‐down of the EMT inducers (Twist1 or Snail) or suppression of the downstream signal (Rac1) attenuates the effect of EMT‐reduced intracellular stiffness. In general, softer cell body in conjunction with better migration capability in EMT may contribute to enhance their ability to invade through blood vessels, and thereby increasing their metastasis potential.

An interesting finding of this study is that when cells were cultivated in conventional 2D cell culture environment, all four HNSCC cells with distinct EMT phenotypes showed no significant differences in intracellular stiffness. Surprisingly, a significant increase in intracellular stiffness was observed in epithelial‐type HNSCC cells (FaDu and CAL‐27), whereas the mesenchymal‐type HNSCC cells (OEC‐M1 and SAS) did not show any significant change. These findings imply that the ECM architectures may play a crucial role in modulating the biophysical and the biochemical characteristics associated with EMT.

In summary, our experimental studies of HNSCC cell lines show a significant difference in the intracellular stiffness of epithelial‐like HNSCC cells versus that of mesenchymal‐like HNSCC cells cultured in 3D environment. A comparison of the intracellular stiffness of HNSCC cells cultured in 2D and 3D revealed that the incremental intracellular stiffness of epithelial—type cells were significantly higher than that of the mesenchymal‐type cells. Our studies also reveal that in 2.5D and 3D cell culture environments, EMT progression is strongly correlated with the invasiveness of the cells and intracellular stiffness; more mesenchymal phenotypes show higher invasiveness and lower intracellular stiffness, whereas more epithelial phenotypes show lower invasiveness and higher intracellular stiffness. Moreover, our results also associate the decreased intracellular stiffness and increased invasiveness with Twist1‐Rac1 pathway or Snail‐mediated pathway. These results jointly support our hypothesis that intracellular stiffness, in conjunction with other cellular biophysical properties such as migration speed and cell morphology, may correlate strongly with the degree of malignant progression, and may thus serve as important complementary metrics in the assessment of the metastatic potential of cancer cells.

**Figure 5:**
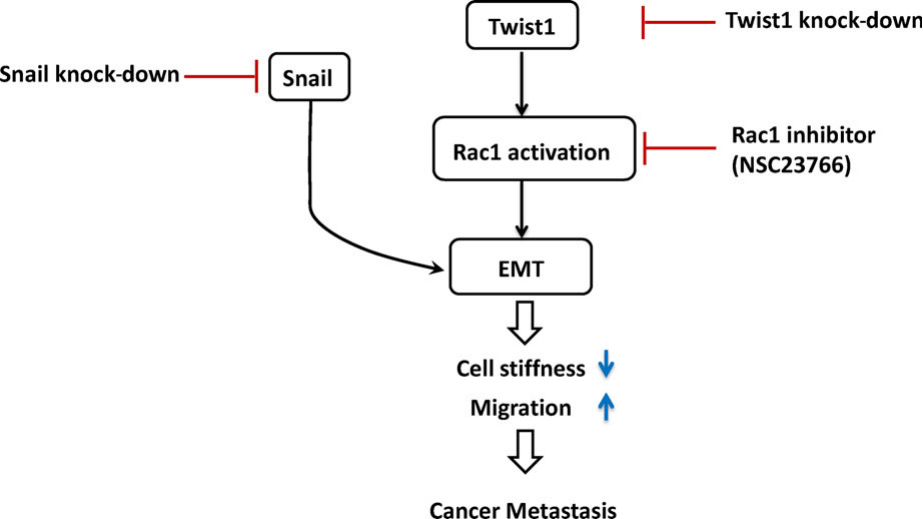
A proposed model of the molecular pathways in EMT, including Twist1‐Rac signal axis and Snail, to regulate cellular migration and invasion capability and intracellular stiffness in EMT in cancer metastasis

Based on these observations, we propose a model of the molecular pathways involving EMT, including Twist1‐Rac signal axis and Snail, to regulate cellular migratory and invasion capability and intracellular stiffness in EMT in cancer metastasis (Figure 5). Our results indicate that both EMT regulators, Snail and Twist1, have a great impact in regulating migration capability and cellular stiffness in 3D system, suggesting the dominant role of EMT in regulating biochemical and biomechanical characteristics of HNSCC cells in 3D microenvironment.

## CONFLICT OF INTEREST

The authors declare that they have no competing financial interests.
