# Supplementary material for: Epithelial‐mesenchymal transition softens head and neck cancer cells to facilitate migration in 3D environments
Source: J Cell Mol Med. 2018 May 4;22(8):3837–46. doi: 10.1111/jcmm.13656 (PMC6050483; doi:10.1111/jcmm.13656)
Supplement: Supplementary file 1 [file JCMM-22-3837-s001.docx]

**Supplementary Materials for**

**Epithelial-mesenchymal transition softens head and neck cancer cells to facilitate migration in 3-demensional environments**

Yin-Quan Chen, Hsin-Yi Lan, Yi-Chang Wu, Wen-Hao Yang, Arthur Chiou, Muh-Hwa Yang


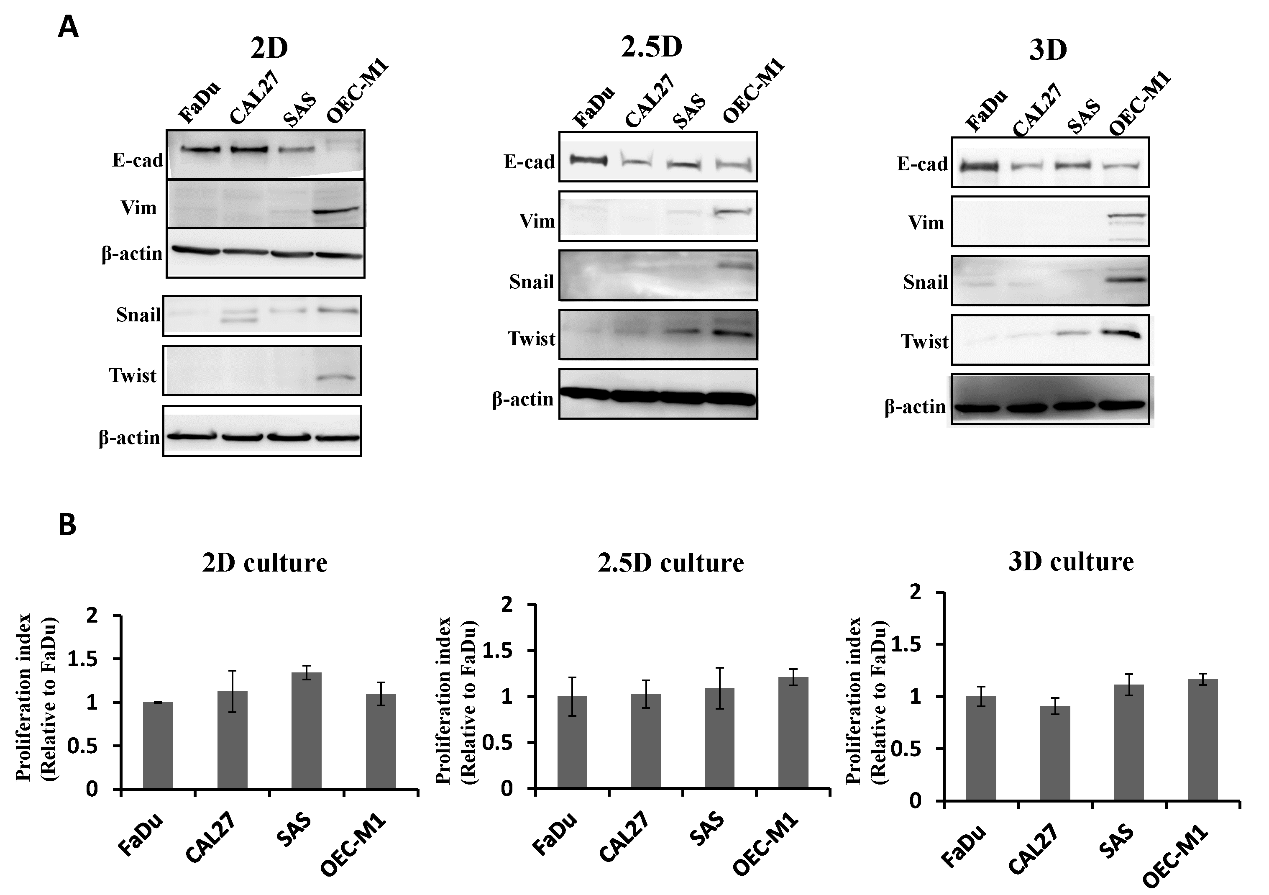


**Figure S1. Analysis of the expression of EMT markers and proliferation index of HNSCC cell lines (FaDu, CAL27, SAS, and OEC-M1) cultured in 2D, 2.5D, and 3D environments for 24hrs. (A)** Respective western blot analysis of E-cadherin (E-cad), vimentin (Vim), Snail, and Twist1 in HNSCC cell lines. β-actin was used as loading control. **(B)** Quantification of the proliferation index of HNSCC cell lines relative to FaDu cells. Data represent mean ± S.E.M. n = 3.


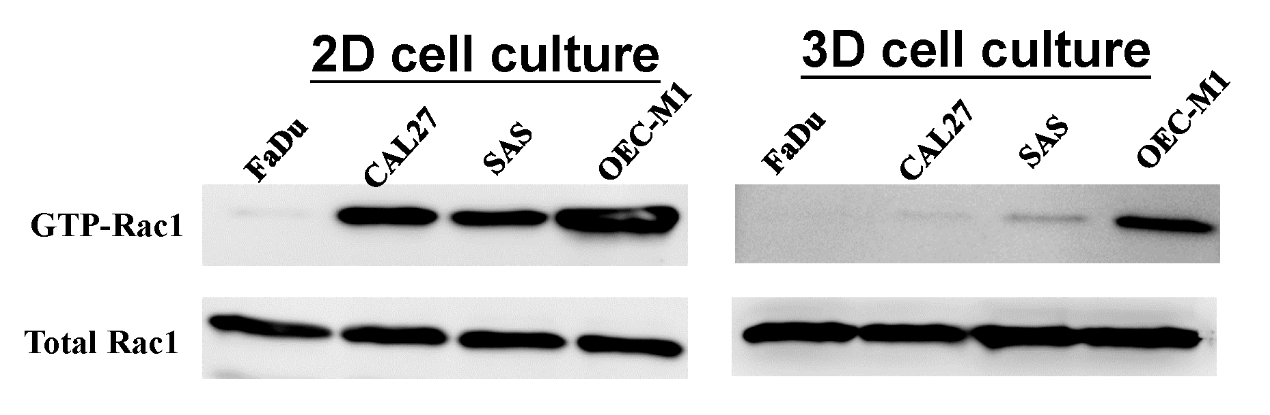


**Figure S2. Immunoblots of pulled down GTP bound Rac1 and total Rac1 in HNSCC cell lines cultured in 2D or 3D environments. Total Rac1 protein was used as the control.**


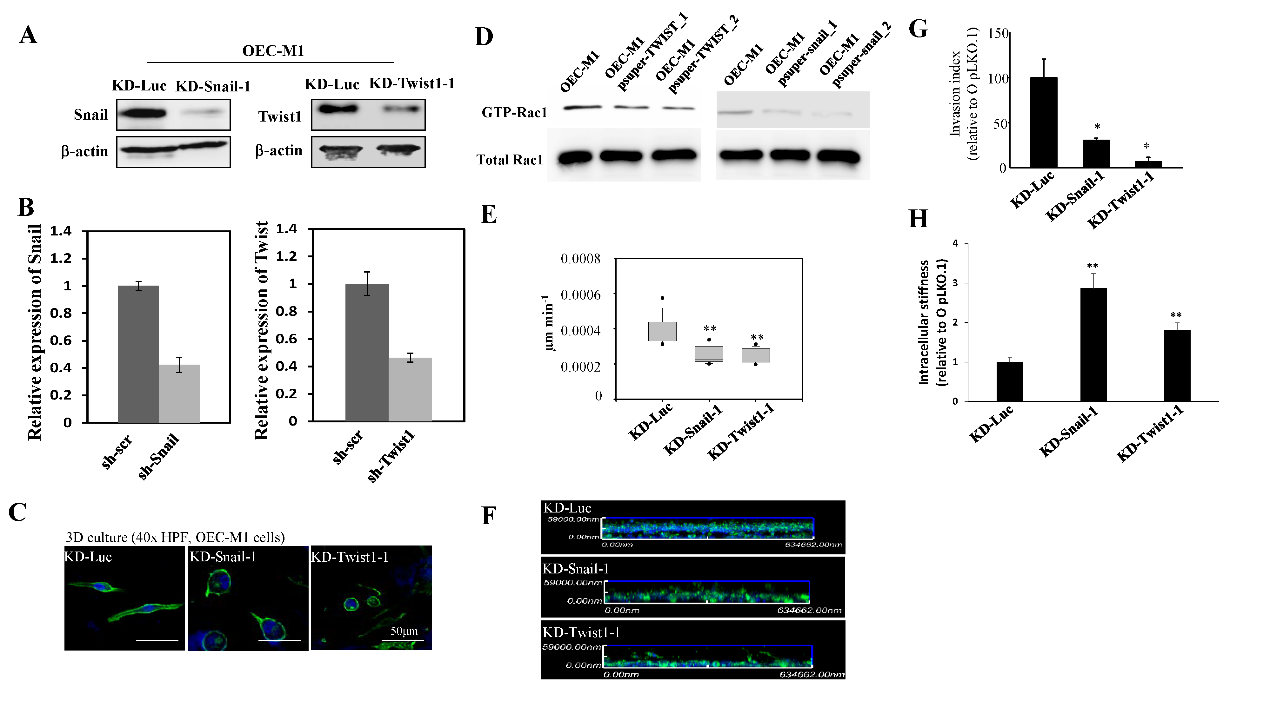


**Figure S3. Knockdown of Snail or Twist1 reduces migration, invasion, and increases intracellular stiffness of OEC-M1 cells. (A)** Western blot of Snail and Twist1 in OEC-M1 cells transfected with the pLKO.1 vector containing a shRNA against Snail (KD-Snail-1), Twist1 (KD-Twist1), or a control sequence. β-actin was a loading control. **(B)** Quantitative RT-PCR analysis of expression of Twist and Snail in OEC-M1 cells transfected with the pSUPER vector containing a shRNA against Snail (sh-Snail), Twist1 (sh-Twist1), or a scrambled sequence (sh-scr). Data represent mean ± S.E.M. n = 2. **(C)** Immunofluorescence to show the morphology and actin organization of OEC-M1 cells receiving KD-Snail-1, KD-Twist1-1, or a control sequence. The cells were embedded in collagen (3D). Green, F-actin; blue, nuclei. Scale bar = 50µm. **(D)** Immunoblots of pulled down GTP bound Rac1 and total Rac1 in OEC-M1 transfected with pSUPER, psuper-TWIST#1, psuper-TWIST#2, psuper-snail#1, and psuper-snail#2. Total Rac1 protein was used as the control. **(E)** Quantification of motility speed of OEC-M1 cells transfected with a shRNA against Snail, Twist1, or a control sequence (n = 10 for each stable cell line). The cell motility speed was calculated and is presented as microns per minute. The box plots represent sample maximum (upper end of whisker), upper quartile (top of box), median (band in the box), lower quartile (bottom of box), and sample minimum (lower end of whisker). **p < 0.01. **(F)-(G)** 3D invasion assay. **(F)** Representative images of OEC-M1 clones invaded into collagen after 24 hr (n = 3). (**G**) Quantification of the invasion index relative to the control clones. Data represents mean ± S.E.M. n = 3. *p < 0.05, **p < 0.01. **(H)** A comparison of the intracellular stiffness (at 10 Hz) of OEC-M1 cells cultured in 3D environment and transfected with a shRNA against Snail, Twist1 vs. a control vector. Data represents mean ± S.E.M. n = 3. **p < 0.01 (relative to the control clone).


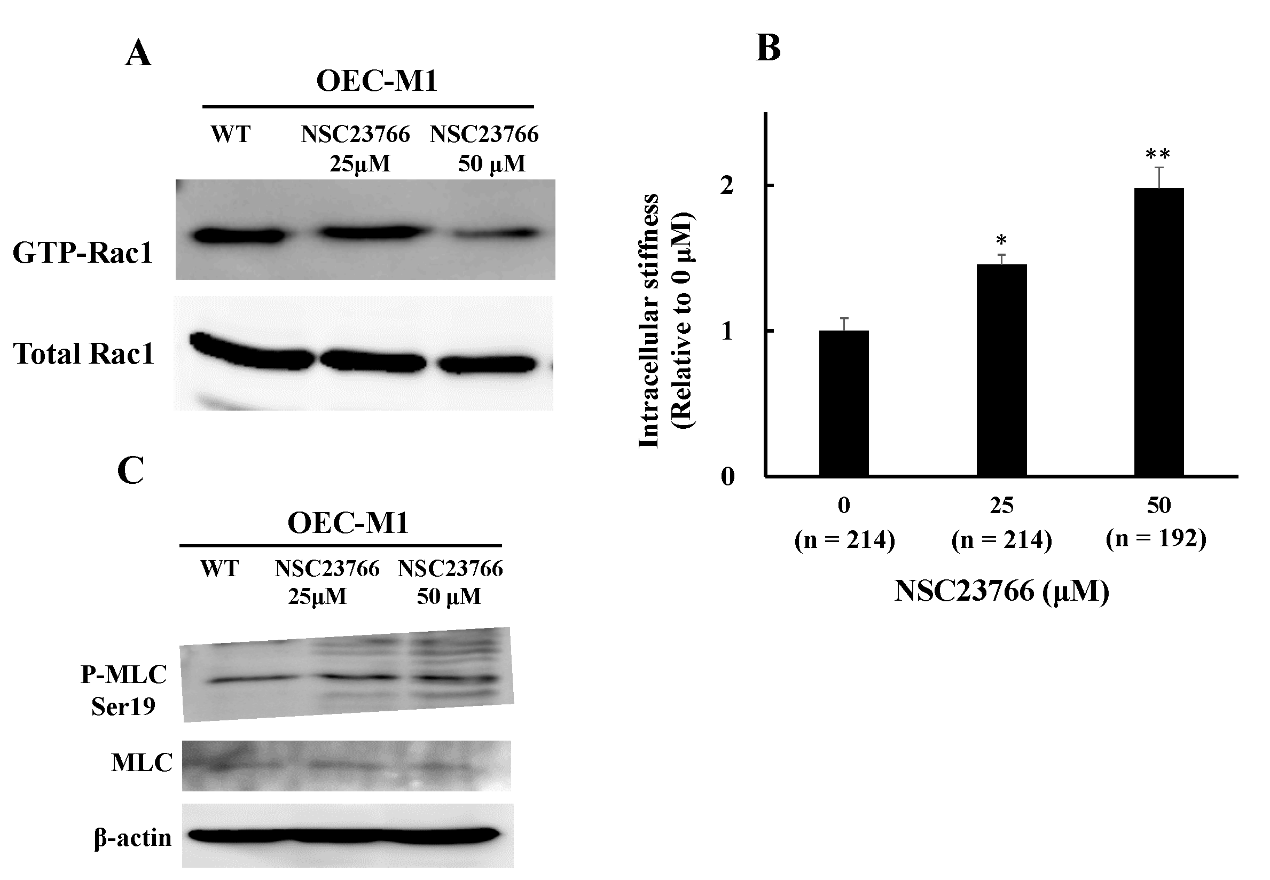


**Figure S4. The effect of Rac1 inhibitor NSC23766 on activation of Rac1, phosphorylation of myosin light chain kinase, and intracellular of OEC-M1 cells. (A)** Immunoblots of pulled down GTP-Rac1 and total Rac1 on OEC-M1 cells treated with 25μM or 50μM of NSC23766 for 24hr. Total Rac1 protein was used as the control. **(B)** The relative intracellular stiffness (at 10Hz) of OEC-M1 cells cultured in 3D environment treated with 25μM and 50μM of NSC23766. Data represent mean ± S.E.M. *p < 0.05; **p < 0.01. **(C)** Western blot of the phosphorylated myosin light chain (MLC) of OEC-M1cells treated with 25μM or 50μM of NSC23766 for 24hr.


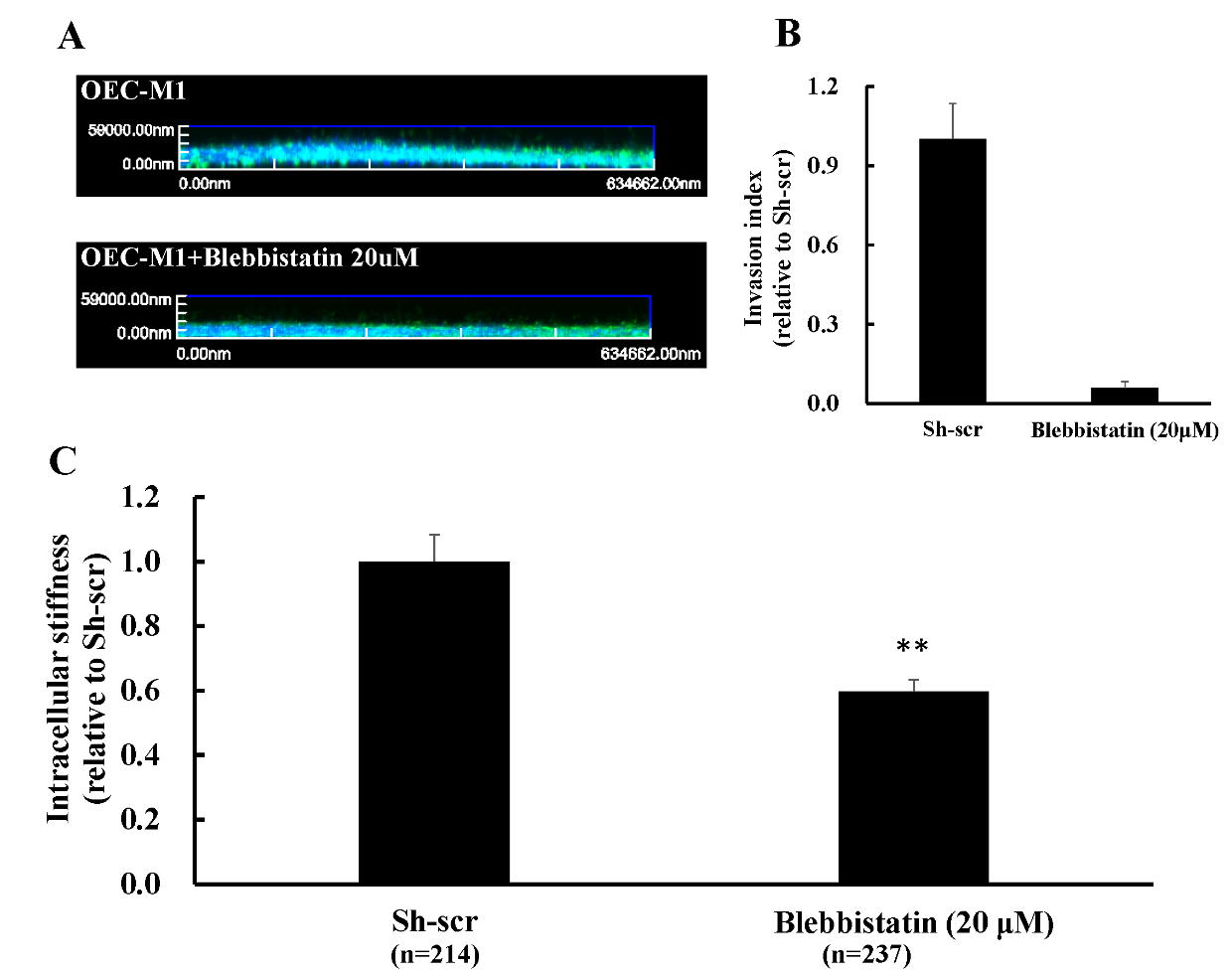


**Figure S5. The effect of Blebbistatin, myosin II inhibitor, on invasion capability and intracellular stiffness of OEC-M1 transfected with the pSUPER (Sh-scr) in 3D environment. (A)** Representative images of OEC-M1 clones invaded into collagen after 24 hr (n = 2). Green: immunofluorescent staining of F-actin of the cells. Blue: nuclei staining. **(B)** Quantification of the invasion index relative to the control clones. Data represent mean ± S.E.M. n = 2. **(C)** A comparison of the intracellular stiffness (at 10 Hz) of OEC-M1 cells cultured in 3D environment and treated 20μM of Blebbistatin relative to the corresponding value of the control cells. Data represent mean ± S.E.M. *p < 0.05; **p < 0.01.


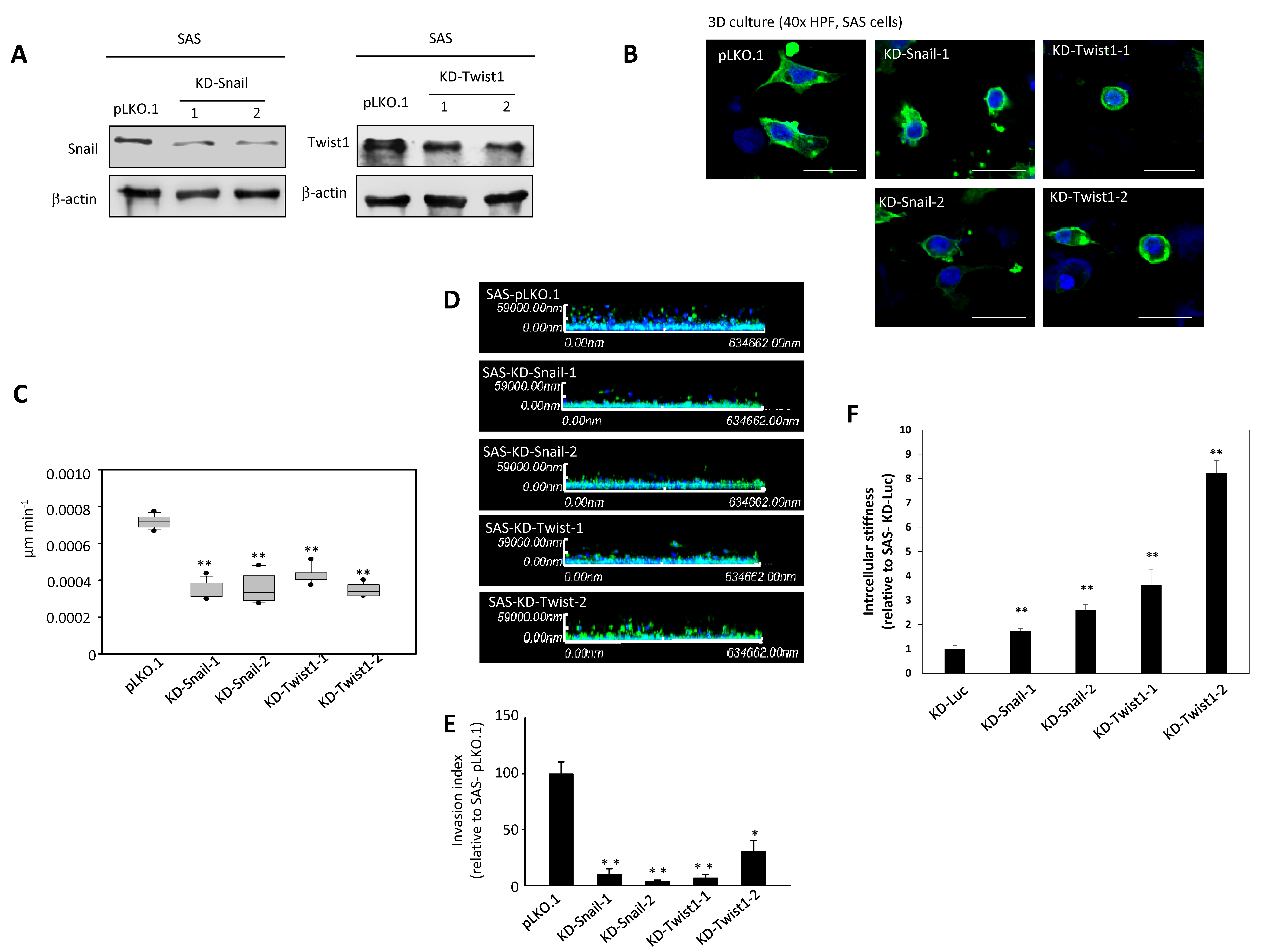


**Figure S6. Knockdown of Snail or Twist1 reduces migration, invasion, and increases intracellular stiffness of SAS cells. (A)** Western blot of Snail and Twist1 in SAS cells transfected with the pLKO.1 vector containing the shRNA sequence against Snail, Twist1, or a control sequence (Luc). Two independent shRNA sequences were used for knocking down each target (KD-Snail-1, KD-Snail-2 for Snail; KD-Twist1-1, KD-Twist1-2 for Twist1). β-actin was a loading control. (**B**) Immunofluorescence to show the morphology and actin organization of SAS cells receiving shRNA against Snail, Twist1, or a control sequence. The cells were embedded in collagen (3D). Green, F-actin; blue, nuclei. Scale bar = 50µm. **(C)** Quantification of motility speed of SAS cells transfected with a shRNA against Snail, Twist1, or a control sequence (n = 10 for each stable cell line). The cell motility speed was calculated and is presented as microns per minute. The box plots represent sample maximum (upper end of whisker), upper quartile (top of box), median (band in the box), lower quartile (bottom of box), and sample minimum (lower end of whisker). **p < 0.01. **(D)-(E)** 3D invasion assay. (**D**) Representative images of SAS clones invaded into collagen after 24 hr (n = 3). **(E)** Quantification of the invasion index relative to the control clones. Data represents mean ± S.E.M. n=3. *p < 0.05, **p < 0.01. **(F)** A comparison of the intracellular stiffness (at 10 Hz) of OEC-M1 cells cultured in 3D environment and transfected with a shRNA against Snail, Twist1 vs. a control vector. Data represents mean ± S.E.M. n = 3. **p < 0.01 (comparing with the control clone).

**

**

**Figure S7**. **A comparison of the relative intracellular stiffness (at 10 Hz) of FaDu cells cultured in 3D environment and transfected with a control vector (FaDu-CMV), a Snail-expressing vector (FaDu-Snail), or a Twist1-expressing vector (FaDu-Twist1). (Normalized to that of the cells treated with CMV).** Data represent mean ± S.E.M. *p < 0.05; **p < 0.01

**Supplementary Table S1. Sequences for shRNA experiments**

| shRNA | clone ID | oligonucleotides sequence (5’ 🡪 3’) |
| --- | --- | --- |
| pSUPER-  sh-scr | N.A. | GATCCCCGTGTCTGTAGGAGTCATCCTTCAAGAGAGGATGACTCCTACAGACACTTTTTA |
| pSUPER-  sh-Snail | N.A. | GATCCCCCACCTCCGGAGATCCTCAATTCAAGAGATTGAGGATCTCCGGAGGTGTTTTTA |
| pSUPER-  sh-Twist1 | N.A. | GATCCCCAGGGCAAGCGCGGCAAGAATTCAAGAGATTCTTGCCGCGCTTGCCCTTTTTTA |
| pLKO.1-  KD-Luc | N.A. | CCTAAGGTTAAGTCGCCCTCGCTCGAGCGAGGGCGACTTAACCTTAG |
| pLKO.1-  KD-Snail-1 | TRCN0000063819 | CCGGCCAGGCTCGAAAGGCCTTCAACTCGAGTTGAAGGCCTTTCGAGCCTGGTTTTTG |
| pLKO.1-  KD-Snail-1 | TRCN0000063821 | CCGGCCAAGGATCTCCAGGCTCGAACTCGAGTTCGAGCCTGGAGATCCTTGGTTTTTG |
| pLKO.1-  KD-Twist1-1 | TRCN0000020540 | CCGGCCTGAGCAACAGCGAGGAAGACTCGAGTCTTCCTCGCTGTTGCTCAGGTTTTT |
| pLKO.1-  KD-Twist1-2 | TRCN0000020542 | CCGGTCCGCAGTCTTACGAGGAGCTCTCGAGAGCTCCTCGTAAGACTGCGGATTTTT |
